# Measurement of population mental health: evidence from a mobile phone survey in India

**DOI:** 10.1093/heapol/czab023

**Published:** 2021-03-09

**Authors:** Diane Coffey, Payal Hathi, Nazar Khalid, Amit Thorat

**Affiliations:** Department of Sociology & Population Research Center, University of Texas at Austin, 305 E 23rd St, RLP 2.602, Austin, TX 78712, USA; r.i.c.e., a research institute for compassionate economics, Delhi, India; Indian Statistical Institute, Delhi Centre, Delhi, India; r.i.c.e., a research institute for compassionate economics, Delhi, India; Department of Sociology, University of California, Berkeley, CA, USA; Department of Demography, University of California, Berkeley, CA, USA; r.i.c.e., a research institute for compassionate economics, Delhi, India; Department of Demography, University of Pennsylvania, Philadelphia, PA, USA; r.i.c.e., a research institute for compassionate economics, Delhi, India; Centre for the Study of Regional Development, Jawaharlal Nehru University, Delhi, India

**Keywords:** Population mental health, India, mobile phone survey, Kessler-6, SRQ

## Abstract

In high-income countries, population health surveys often measure mental health. This is less common in low- and middle-income countries (LMICs), including in India, where mental health is under-researched relative to its disease burden. The objective of this study is to assess the performance of two questionnaires for measuring population mental health in a mobile phone survey. We adapt the Kessler-6 screening questionnaire and the World Health Organization’s Self-Reporting Questionnaire (SRQ) for a mobile phone survey in the Indian states of Bihar, Jharkhand and Maharashtra. The questionnaires differ in the symptoms they measure and in the number of response options offered. Questionnaires are randomly assigned to respondents. We consider a questionnaire to perform well if it identifies geographic and demographic disparities in mental health that are consistent with the literature and does not suffer from selective non-response. Both questionnaires measured less mental distress in Maharashtra than in Bihar and Jharkhand, which is consistent with Maharashtra’s higher human development indicators. The adapted SRQ, but not the adapted Kessler-6, identified women as having worse mental health than men in all three states. Conclusions about population mental health based on the adapted Kessler-6 are likely to be influenced by low response rates (about 82% across the three samples). Respondents were different from non-respondents: non-respondents were less educated and more likely to be female. The SRQ’s higher response rate (about 94% across the three states) may reflect the fact that it was developed for use in LMICs and that it focuses on physical, rather than emotional, symptoms, which may be less stigmatized.

**Key Messages**

- Increasing mobile phone ownership makes mobile phone surveys a potentially valuable medium for measuring population mental health in low- and middle-income countries.
- Adaptations to simplify the Kessler-6 and the self-reporting questionnaires (SRQs) aided measurement of mental health among mobile phone survey respondents in India.
- Compared to the adapted Kessler-6, the adapted SRQ yielded higher response rates and more consistently identified gender differences in mental health. Both questionnaires identified regional disparities in mental health that are consistent with community studies.

## Introduction

In high-income countries, population health surveys often include questions on mental health in addition to physical health. For example, the National Health Interview Survey conducted by the United States Centers for Disease Control and Prevention developed the Kessler-6 scale to measure psychological distress in the U.S. population (Kessler *et al.*, 2003). The questions were later adapted for use in other settings (Kessler *et al.*, 2010). The Kessler scale and other tools to measure mental health, like the Center for Epidemiological Studies Depression Scale (CES-D), the Generalized Anxiety Disorder (GAD) scale and the Patient Health Questionnaire (PHQ), can screen for common mental disorders in clinical settings (Patel *et al.*, 2008; Kumar *et al.*, 2016; van Heyningen, 2018). They have also contributed to tracking trends in mental health in populations and to describing disparities in mental health by population groups (Perreira *et al.*, 2005; Rosenfield and Mouzon, 2013; Case and Deaton, 2017).

Despite the high burden of mental disorder in low- and middle-income countries (LMICs) (Votruba et al., 2020) and the fact that the World Health Organization’s (WHO’s) self-reporting questionnaire (SRQ) has been shown to be suitable for administration by lay interviewers (Harpham *et al.*, 2003), measurement of mental health in population health surveys is less common outside of high-income countries. The Demographic and Health Surveys’ (DHS) Model Questionnaires, for example, cover reproductive and child health, anthropometry, HIV, anaemia, malaria and chronic disease but not mental health (Demographic and Health Survey Program, 2019). Governments and international organizations might reasonably prioritize measuring physical health where mortality rates are high and infectious disease is widespread. However, as mortality and fertility rates decline and as data become less costly to collect, there are emerging opportunities to measure, understand and address poor mental health in LMIC populations.

The project of measuring population mental health in LMIC may be facilitated by the use of mobile phone surveys, which are less costly than face-to-face surveys (Pinto-Meza *et al.*, 2005).^1^ Indeed, mobile phone technology is increasingly used to deliver health information in poor countries, and the COVID-19 pandemic has led to increased use of phone surveys (Pop-Eleches *et al.*, 2011; Bastawrous *et al.*, 2013; Himelein *et al.*, 2020). Although mobile phone surveys would have been unreliable for measuring population mental health in developing countries previously (Harpham *et al.*, 2003), increasing mobile phone ownership means that it may now be possible to do so in representative samples (Leo *et al.*, 2015), as done in developed countries (Kroenke *et al.*, 2009).^2^ India’s DHS finds that household-level mobile phone coverage increased dramatically from 17% in 2005 to 90% in 2015.

This article advances the literature on the measurement of population mental health in LMICs by testing two mental health questionnaires in a mobile phone survey conducted in three states of India. In particular, we evaluate adaptations of the Kessler-6 and the SRQ. The adaptations, described below, made these questionnaires suitable for use in a mobile phone survey.

Because no prior survey uses these adaptations of the Kessler-6 and the SRQ, and because no prior survey measures mental health in a phone survey, we are not able to make comparisons of our results outside of the data that we collected.^3^ Instead, we assess the performance of these two questionnaires by qualitatively comparing their results to what we would expect based on prior research on geographic and demographic variation in mental health in India. Research suggests that on average, women have worse mental health than men (Mumford *et al.*, 1997; Patel *et al.*, 1999; Poongothai *et al.*, 2009; Das *et al.*, 2012; Anand 2015), less-educated people have worse mental health than more-educated people (Hackett *et al.*, 2007) and poorer people have worse mental health than richer people (Fahey *et al.*, 2016).

The article proceeds as follows. In the Materials and Methods section, we first describe the setting in which the study was conducted; we then describe the data source: the Social Attitudes Research, India (SARI) mobile phone survey; finally, we describe how we adapted the questionnaires for use in a mobile phone survey and how we analyse the data. In the Results section, we present response rates, summary statistics and characteristics of non-response for each questionnaire; we also show the results of ordered logit regressions that describe demographic correlates of poor mental health as measured by each questionnaire. In the Discussion section, we provide interpretation of the Results, and discuss limitations of the study and directions for future research. The findings of this research support the conclusion that sufficiently simple mental health questions, such as those in the adapted SRQ, can be usefully employed to measure population mental health in phone surveys where representative sampling can be achieved.

## Materials and methods

### The study setting

#### The states of Bihar, Jharkhand and Maharashtra

Data for this study were collected in the Indian states of Bihar, Jharkhand and Maharashtra. Until 2000, Bihar and Jharkhand were one state. Bihar and Jharkhand have similar geographic sizes but very different population sizes: the 2011 population of Bihar was about 100 million, compared to 32 million in Jharkhand. The 2011 population of Maharashtra was approximately 112 million in a land area three times as large as Bihar.

Maharashtra is different from Bihar and Jharkhand on a number of health and socioeconomic indicators. India’s 2017 Sample Registration System (SRS) Statistical Report reports infant mortality in Maharashtra at 19 per 1000 live births, compared with 35 in Bihar and 29 in Jharkhand. The SRS reports a total fertility rate in Maharashtra of 1.7, while it is 3.2 in Bihar and 2.5 in Jharkhand. According to India’s most recent DHS, the 2015/16 National Family Health Survey (NFHS), the average adult in Maharashtra has 7.6 years of education, but only 4.8 years in Bihar and 5.6 years in Jharkhand. Furthermore, the 2011 Indian Census reported a literacy rate for Maharashtra of 83%, compared to 64% in Bihar and 68% in Jharkhand. India’s Ministry of Statistics and Program Implementation reports the 2018–19 annual net state domestic product per capita to be ₹191 736 for Maharashtra, compared to ₹43 822 for Bihar and ₹76 019 for Jharkhand (MoSPI, 2019).

The states also differ by the prevalence of caste and gender discrimination. The fraction of upper-caste adults who reported practicing untouchability—a severe form of discrimination against people from lower castes^4^—in the SARI survey (described below) was 25% [95% confidence interval (CI): 21%, 30%] in Maharashtra, compared to 48% (95% CI: 45%, 51%) in Bihar, and 37% (95% CI: 31%, 44%) in Jharkhand. One important measure of household-level gender discrimination in India is whether women eat meals only after men have finished eating. SARI finds that women usually eat last in 31% (95% CI: 28%, 34%) of households in Maharashtra, compared to 70% (95% CI: 68%, 72%) of households in Bihar and 53% (95% CI: 48%, 58%) in Jharkhand.

#### Evidence from prior studies on correlates of poor mental health in India

This study measured mental health as mental distress using adaptations of the Kessler-6 and the SRQ. The focus on mental distress is valuable because it measures mental health on a continuum, rather than by identifying people as suffering from a mental disorder or not. This approach has the disadvantage of making it more difficult to compare the findings with the published literature, which tends to focus on mental disorders. For example, the 2015–16 National Mental Health Survey collected data on mental disorders across 12 states from 39 532 individuals (Gururaj *et al.*, 2016). This survey was an important input into a recent meta-analysis of state-level variation in mental disorders in India (Sagar *et al.*, 2020). In contrast to patterns in the SARI data, the Sagar *et al.* (2020) meta-analysis finds a higher prevalence of mental disorders in states with higher levels of development, as well as an increasing prevalence of mental disorders over time. Both of these findings are consistent with the idea that capacity for diagnosing mental disorder may be an important reason for the higher prevalence of mental disorders. Measuring mental distress with simple questions provides a valuable complement to measuring mental disorder, especially while capacity for diagnosis of mental health disorders remains low in parts of India.

Community studies from India and other LMICs may provide the best indication of what associations between mental health and demographic and socioeconomic indicators we should expect from a mental health questionnaire. We expect to see better mental health among men compared to women: across countries, gender disadvantage of multiple forms, including limited access to resources, restricted choices and discrimination have been shown to have negative effects on mental health for women (Chandra and Satyanarayana, 2010; [Bibr czab023-B22][Bibr czab023-B23]). We also expect that lower education and fewer assets will have a negative correlation with mental health outcomes. A lack of education may be an indication of childhood adversity, low social status or a lack of opportunity, which may in turn hurt mental health (Araya *et al.*, 2003). Poverty may put individuals at greater risk of developing mental health disorders because of social exclusion, high levels of stress and higher likelihood of experiencing adverse events that lead to insecurity (Patel and Kleinman, 2003; Das *et al.*, 2007; Lund *et al.*, 2010).

Evidence is also emerging that low-caste groups and Muslims, India’s largest minority religion, have worse mental health than individuals from higher-status groups (Gupta and Coffey, 2019). A study from Uttarakhand, a state in north India, found that low-caste individuals were more likely to report having depression than high-caste individuals (Mathias *et al.*, 2015). And in a study of five north-Indian states, Spears (2016) finds that Dalits, those of the lowest caste, report worse life satisfaction than any other caste, even controlling for education and asset wealth.

We expect that people in Maharashtra will experience less mental distress, on average, than people in Bihar and Jharkhand because Maharashtra fares much better across socioeconomic characteristics, health statistics and the extent of gender and caste discrimination.

### The SARI survey

We use data from the SARI survey. SARI is a mobile phone survey designed to measure attitudes towards marginalized groups, including women, lower castes and Muslims, and to measure opinions about public policies in India. Prior to collecting data in Bihar, Jharkhand and Maharashtra, SARI collected data in Delhi and Uttar Pradesh (2016), and Rajasthan and Mumbai (2017). The Bihar, Jharkhand and Maharashtra samples introduced questions on health—particularly on abortion (Broussard *et al.*, 2019) and mental health—to the SARI survey.

The SARI survey builds representative samples of adults ages 18–65 in both rural and urban areas by using probability-weighted random digit dialling and within-household respondent selection. Specifically, we provide interviewers a list of phone numbers: The first five digits are codes that the Telecom Regulatory Authority of India (TRAI) issues to mobile phone companies based on the geographic mobile circle from which the number originates, and the last five digits are randomly generated. The number of times a particular five-digit code appears in the list is proportional to the number of subscriptions that mobile companies report to TRAI.

SARI interviewers call these phone numbers in a random order and speak to respondents of the same sex to make respondents comfortable. Once a respondent of the interviewer’s same sex agrees to participate, they are asked to list all adults of their sex in the household. Survey respondents are selected randomly from the household listing by Qualtrics software to ensure (i) that even individuals who do not own their own mobile phones are eligible to be interviewed and (ii) that even the least educated adults, who may be less likely to participate in a phone survey, are represented in our sample.

Table 1 shows SARI sample sizes and response rates by state. Although SARI’s response rates may appear low compared to response rates typically seen in face-to-face interviews, they are quite high compared with phone surveys in other countries. A Pew Research Center study from the USA (Kohut *et al.*, 2012) found an average response rate of 9% in its 2012 surveys. They concluded that weighting phone survey data to match the demographic composition of the population can sufficiently adjust for low response rates and that phone surveys can provide accurate estimates of public opinion. SARI’s sample sizes are consistent with other representative samples used to analyse social attitudes.

**Table 1:** SARI sample sizes and response rates, by state

State	Sample sizes			Response rates (%)
	Men	Women	Total	
Bihar	1450	1988	3438	19
Jharkhand	459	550	1009	
Maharashtra	920	746	1666	25
Total	2829	3284	6113	

*Note*: Survey response rates are calculated as the number of surveys in which a respondent answered at least a third of the questions divided by the number of mobile numbers that were valid (as opposed to non-existent, switched off or not available) when they were first called. Response rates for Bihar and Jharkhand cannot be calculated separately because Bihar and Jharkhand mobile numbers are pooled into the same mobile circle by the Telecom Regulatory Authority of India. State of residence is only known for individuals who began the survey, but not for every valid phone number called.

NFHS-2015 data suggest that low mobile phone ownership is unlikely to present a major obstacle to achieving a representative sample in this context. Table 2 shows the fraction of households in each state that own a mobile phone. Coverage in Bihar and Maharashtra is similar at approximately 90% and 91%, respectively, while coverage in Jharkhand is lower, at 84%. Urban areas across all three states have higher coverage than rural areas, with the greatest urban-rural difference in Jharkhand.

**Table 2:** Household-level mobile phone ownership, by state

State	Urban (%)	Rural (%)	Total (%)
Bihar	95	89	90
Jharkhand	95	80	84
Maharashtra	97	86	91
Total	96	87	90

*Note*: Data source: National Family Health Survey, 2015–16.

Table 3 shows the distributions of demographic characteristics among households in the NFHS-2015 that do and do not own a mobile phone. In each of the three states, Scheduled Caste households, and those which do not have electricity or use a latrine are over-represented among households that do not own mobile phones. To the extent that these characteristics are correlated with poor mental health, SARI may underestimate the prevalence of poor mental health. However, when considering the costs and benefits of using mobile phones to measure mental health, it is important to remember that mobile phone coverage may have improved substantially since 2015. For example, in Bihar, where data were collected over a 6-month period in 2015, households interviewed in the last month of the survey were 4 percentage points more likely to own a mobile phone than households interviewed in the first month.

**Table 3:** Characteristics associated with household mobile phone ownership in NFHS-2015, weighted

	Bihar		Jharkhand		Maharashtra	
	Does not own phone	Owns phone	Does not own phone	Owns phone	Does not own phone	Owns phone
Caste of household head						
Proportion Scheduled Caste	0.299	0.197	0.169	0.137	0.213	0.174
	(0.011)	(0.005)	(0.010)	(0.005)	(0.013)	(0.007)
Proportion Scheduled Tribe	0.050	0.033	0.474	0.242	0.280	0.097
	(0.012)	(0.002)	(0.016)	(0.009)	(0.022)	(0.005)
Proportion OBC	0.537	0.586	0.320	0.493	0.223	0.286
	(0.012)	(0.006)	(0.013)	(0.009)	(0.013)	(0.008)
Proportion other	0.115	0.183	0.038	0.128	0.284	0.442
	(0.008)	(0.005)	(0.004)	(0.006)	(0.018)	(0.011)
Religion of household head						
Proportion Hindu	0.835	0.838	0.715	0.745	0.798	0.785
	(0.011)	(0.007)	(0.015)	(0.010)	(0.016)	(0.010)
Proportion Muslim	0.163	0.161	0.069	0.142	0.076	0.110
	(0.011)	(0.007)	(0.007)	(0.009)	(0.014)	(0.009)
Proportion other	0.002	0.001	0.217	0.114	0.126	0.105
	(0.000)	(0.001)	(0.015)	(0.006)	(0.010)	(0.006)
Household electricity						
Proportion without electricity	0.686	0.385	0.454	0.150	0.276	0.056
	(0.010)	(0.008)	(0.013)	(0.007)	(0.014)	(0.004)
Proportion with electricity	0.314	0.615	0.546	0.850	0.724	0.944
	(0.010)	(0.008)	(0.013)	(0.007)	(0.014)	(0.004)
Household latrine						
Proportion that does not use one	0.895	0.648	0.922	0.660	0.640	0.261
	(0.006)	(0.006)	(0.006)	(0.009)	(0.017)	(0.009)
Proportion that uses one	0.105	0.352	0.077	0.340	0.360	0.739
	(0.006)	(0.006)	(0.006)	(0.009)	(0.017)	(0.009)
Mean years of household head education	1.90	4.84	2.21	5.97	3.09	7.45
	(0.07)	(0.06)	(0.07)	(0.07)	(0.14)	(0.11)

*Note*: The table shows the distributions of demographic and socioeconomic characteristics among households that do and do no own mobile phones in the NFHS-2015. Survey weights are used to compute the distributions.

Even if household-level mobile phone ownership is now near-universal, less educated or rural adults may still be underrepresented if they are less likely to keep their phones on or less likely to agree to participate in the survey. Supplementary Figure S1A shows distributions of education among adults in the SARI survey and in the 2011 Census. It shows that SARI under-represented less educated respondents in the raw sample.

To account for different response rates of demographic groups, we construct and use survey weights based on the sex, age, place and education distributions of the population. Sample statistics are representative of the state population if, conditional on sex-by-age-by-education bins, respondents’ answers are similar to answers that would have been given by people who were not reached or who refused. All of the results presented in the paper use these survey weights.

To improve the quality of the sample and to reduce social desirability bias, interviewers interview respondents of the same sex. To reduce non-sampling errors, interviewers use caste- and religion-neutral names, refrain from showing approval or disapproval for respondent answers, and take care to explain the study’s purpose. Coffey *et al.* (2018) assess the quality of SARI data by comparing it to data from the Indian Human Development Survey (IHDS), a face-to-face survey of over 40 000 households: SARI’s state-level estimates of practices of discrimination against women and Dalits are not statistically distinguishable from the IHDS’, which points to the high quality of the SARI data.

SARI data and documentation are publicly available. More information about SARI’s phone survey methods is available in Hathi *et al.* (2020a,b).

### Adaptations of Kessler-6 and self-reporting questionnaires

The Kessler-6 questionnaire was developed for use in the USA. It was designed to measure psychological distress based on answers to six questions related to a respondent’s emotional state (Kessler *et al.*, 2003). The Kessler-6 has been validated through the World Mental Health Survey Initiative for LMIC contexts (Kessler *et al.*, 2010; Tesfaye et al., 2010), confirming that responses to the Kessler-6 match well with independent clinical assessments of mental illness.

For each question, respondents are asked to report whether, in the 30 days prior to the interview, they experienced a negative feeling all of the time, most of the time, some of the time, a little of the time or none of the time. Each question is scored from 5 to 0, with higher numbers indicating worse mental health. Because there are six questions, the range of possible scores is from 0 to 30.

The SRQ was developed by the WHO for use by primary health workers with limited training in LMIC settings (Beusenberg and Orley, 1994). It includes 20 questions that focus on physical symptoms that are easy to understand, and a ‘yes’ or ‘no’ response format. Researchers have adapted the SRQ to a variety of settings and have validated its ability to assess mental health across cultural contexts with reasonable accuracy (de Jesus Mari and Williams, 1986; Husain *et al.*, 2006; Youngmann et al., 2008; Chen *et al.*, 2009).

Respondents to the SARI survey were randomly assigned to receive either an adapted Kessler-6 questionnaire or an adapted SRQ. No respondent answered both sets of mental health questions. We describe the adaptations to these questions here. Both sets of mental health questions appeared after questions on asset and latrine ownership.

SARI interviewers introduced Kessler-6 questions with the following text: ‘We do not always feel the same way. Sometimes we are sad and sometimes we are happy, sometimes we are worried and sometimes relaxed. In the next few questions, I will ask how you have been feeling in the past one month’. This is a slight elaboration on the original text: ‘The next questions are about how you have been feeling in the past 30 days’. Our experiences of piloting the Kessler-6 suggested a longer introduction was useful because respondents were confused when the interviewer abruptly began asking about their feelings after asking about household assets.

The original (unadapted) Kessler-6 questions are listed in Table 4. When the Kessler-6 is administered verbally, the interviewer reminds the respondent of the 30-day reference period and the five answer options for each question.

**Table 4:** Original questions asked in Kessler-6 and self-reporting questionnaires

Kessler-6 questionnaire	Self-reporting questionnaire
About how often during the past 30 days did you feel ‘nervous’—would you say all of the time, most of the time, some of the time, a little of the time or none of the time? About how often during the past 30 days did you feel ‘hopeless’—would you say all of the time, most of the time, some of the time, a little of the time or none of the time? About how often during the past 30 days did you feel ‘restless or fidgety’—would you say all of the time, most of the time, some of the time, a little of the time or none of the time? About how often during the past 30 days did you feel ‘so depressed that nothing could cheer you up’—would you say all of the time, most of the time, some of the time, a little of the time or none of the time? About how often during the past 30 days did you feel ‘that everything was an effort’—would you say all of the time, most of the time, some of the time, a little of the time or none of the time? About how often during the past 30 days did you feel ‘worthless’—would you say all of the time, most of the time, some of the time, a little of the time or none of the time?	Do you often have headaches? Is your appetite poor?* Do you have trouble sleeping?* Are you easily frightened? Do your hands shake? Do you feel nervous, tense or worried? Is your digestion poor? Do you have trouble thinking clearly?* Do you feel unhappy? Do you cry more than usual? Do you find it difficult to enjoy your daily activities? Do you find it difficult to make decisions?* Is your daily work suffering? Are you unable to play a useful part in life? Have you lost interest in things? Do you feel that you are a worthless person? Has the thought of ending your life been on your mind?* Do you feel tired all the time?* Do you have uncomfortable feelings in your stomach? Are you easily tired?

*Note*: Source for Kessler-6 questionnaire: National Comorbidity Survey: https://www.hcp.med.harvard.edu/ncs/k6_scales.php. Source for SRQ: A User’s Guide to the Self-Reporting Questionnaire (Beusenberg and Orley, 1994).

* indicates that a question was included in the SARI survey.

We initially piloted Hindi translations of the Kessler-6 questions in a face-to-face setting. After revising the translation, we piloted the questions by phone. Many respondents were not able to keep track of five response options, which led to high rates of non-response. However, when the number of response options was reduced to three, more respondents were able to answer. Therefore, the SARI survey maintained the same questions asked by the Kessler-6 but adapted the answer options from the five described above to three: ‘always’, ‘sometimes’ or ‘never’. Whereas studies that use the five-option scale often present results on a scale of 0 to 24, our results for the adapted Kessler-6 are on a scale of 0 to 12 possible points, where lower numbers represent better mental health.

In SARI, the SRQ questions were introduced with the following text: ‘In the next few questions, I will ask you about the sadness or problems you may have faced in the last 30 days. If something like this happened in the last 30 days, say yes. If this did not happen in the last 30 days, say no. Now I will ask you questions one-by-one’. This is the same text as is recommended by Beusenberg and Orley (1994) in the *User’s Guide to the Self Reporting Questionnaire* published by the WHO.

Similar to the Kessler-6 questions, Hindi translations of the SRQ were first piloted face-to-face and then by phone. Over the phone, many respondents became confused or frustrated by the similarity across the SRQ questions. To reduce attrition and to achieve a closer comparison with the Kessler-6 Questionnaire, we included six out of the original 20 SRQ questions in our adapted SRQ Questionnaire. The questions we chose focused on physical (rather than emotional) experiences to provide a contrast to the way that the Kessler-6 assesses mental health. Table 4 lists the full set of SRQ questions. Those used in SARI are marked with an asterisk. We hypothesized that respondents might more readily talk about what they saw as physical experiences, rather than about emotional ones. Although the literature has validated self-reports of physical symptoms as a way of assessing mental health (Tylee and Gandhi, 2005; Kapfhammer, 2006), our respondents may not have known that these questions were intended to measure mental health.

Respondents who were assigned the SRQ typically answered the questions more easily than those who were assigned the Kessler-6 questionnaire. Our experiences from piloting and speaking with interviewers suggest that this is in part because of the SRQ’s ‘yes’ or ‘no’ answer format.

### Analysis of response rates

To analyse response rates for the two questionnaires, we compute weighted proportions of people who answered all of the mental health questions, some and none. Respondents who answered the asset section prior to the mental health section are considered eligible to answer mental health questions. They are included in the denominator for computing the response rate. Respondents who began the survey but stopped participating before the household asset section are not included in the denominator. Unfortunately, we do not have data about participants who declined to participate in the survey entirely.

### Analysis of selection into non-response

To examine whether respondents with certain characteristics are more likely not to respond to each set of mental health questions, we present two analyses. First, we use single-variable logit models to regress an indicator for non-response on demographic characteristics, separately for respondents who were assigned to each questionnaire. For this analysis, we combine data from all three states and use pooled weights. The model we estimate is:

\begin{equation*} logit\left( {non - respons{e_i}} \right) = {\beta _1}demographic\;characteristi{c_i} + {\varepsilon _i}, \end{equation*}

where *i* indexes the individual. The dependent variable is whether a respondent left all or some of the mental health questions unanswered. We run separate regressions for the following independent variables: (i) whether the respondent is female, (ii) whether the respondent is over age 45, (iii) whether the respondent has <9 years of education and (iv) whether the respondent owns two or fewer assets. We present the results as odds ratios.

Second, we present the marginal effects at the mean from a logistic regression that interacts all of the independent variables described above. This approach has the advantage of telling us what the difference in probability of non-response would be if we took an otherwise average individual, and made that person female rather than male, over age 45 rather than younger, etc.

### Analysis of mental health outcomes

For both the adapted Kessler-6 questionnaire and the adapted SRQ, the primary measure of mental health that we analyse is a mental health score. For the adapted Kessler-6, respondent mental health scores range from 0 to 12, as described above. For the adapted SRQ, the mental health score is the sum of indicator variables for having answered ‘yes’ to an SRQ question. Therefore, the SRQ scores range from 0 to 6.

We examine predictors of mental health score for each questionnaire in order to assess the quality of the questions in this context.

The mental health scores are ordered variables; therefore, we analyse the correlates of poor mental health using ordered logit regression. In an ordered logit model, a latent variable m* is assumed to be a linear function of the independent variables, with an error term with a logistic distribution. The ordered outcome ‘categories’ correspond to ‘cut-points’ in the continuous distribution of m* that are unobservable parameters fit by maximum likelihood (Rodriguez, 2007). Ordered logit regression analysis allows us to investigate which characteristics predict mental health among respondents from each questionnaire. One disadvantage of the ordered logit approach, however, is that it constrains the covariates to have the same linear effect on latent mental health at each cut point.

\begin{equation*} m_i^* = {\beta _1}femal{e_i} + Age\;grou{p_i}\Theta + Education\;grou{p_i}\Gamma + {\beta _2}Musli{m_i} + Caste\;grou{p_i}\Lambda + {\beta _3}count\;of\;asset{s_i} + {\varepsilon _i} \end{equation*}

where *ɛ_i_* has a logistic distribution and the ordered logit link function additionally includes cut-points for levels of the outcome variable. Subscripts *i* index respondents. *female_i_* is an indicator for whether person *i* is female; *Age group_i_* is a set of four dummy variables for the age of the respondent, in years; *Education group_i_* is a set of four indicators for educational attainment; *Muslim_i_* is an indicator for being Muslim; *Caste group_i_* is a set of five indicators for whether a respondent is Scheduled Caste, Other Backward Class, Scheduled Tribe, general caste or Brahmin; *count of assets_i_* is the number of assets (out of five) that the respondent’s household owns. The assets that the SARI survey asks about are mixers, scooters, fans, refrigerators and pressure cookers. We do not show separate coefficients for each caste group because some caste groups are quite small. Instead, we show the results of an *F*-test of the statistical significance of all caste indictors in predicting mental health score.

### Ethical approval

IRB approval for SARI data collection was obtained under protocol #16-003. Surveys were conducted by phone. Oral consent was obtained because surveyors did not meet the respondents in person. Consent was documented in Qualtrics software.

## Results

### Summary statistics about respondents

Table 5 summarizes respondent characteristics. Summary statistics are reported by state and by mental health questionnaire. There are differences in schooling and asset ownership across states: respondents in Bihar and Jharkhand are less educated than those in Maharashtra and own fewer assets, on average. Bihar and Jharkhand also have higher proportions of Muslim and lower caste respondents than Maharashtra. There are not meaningful differences in the characteristics of respondents who answered each type of questionnaire because questionnaires were randomly assigned to respondents. Any differences in measured mental health across questionnaires can be attributed to differences in the questionnaire rather than to differences in respondent characteristics.

**Table 5:** Summary statistics for predictors of mental health score, by state

	Bihar				Jharkhand				Maharashtra				Total			
	Kessler		SRQ		Kessler		SRQ		Kessler		SRQ		Kessler		SRQ	
	Prop.	S.E.	Prop.	S.E.	Prop.	S.E.	Prop.	S.E.	Prop.	S.E.	Prop.	S.E.	Prop.	S.E.	Prop.	S.E.
Female	0.46	0.01	0.46	0.01	0.50	0.03	0.50	0.02	0.47	0.02	0.48	0.02	0.47	0.01	0.47	0.01
Education																
No schooling	0.47	0.01	0.46	0.01	0.39	0.03	0.47	0.02	0.24	0.02	0.23	0.02	0.34	0.01	0.35	0.01
1–8 years schooling	0.26	0.01	0.28	0.01	0.32	0.02	0.22	0.02	0.36	0.02	0.35	0.02	0.32	0.01	0.30	0.01
9–12 years schooling	0.19	0.01	0.17	0.01	0.19	0.02	0.19	0.02	0.26	0.02	0.28	0.02	0.23	0.01	0.22	0.01
13+ years schooling	0.08	0.01	0.09	0.01	0.10	0.02	0.12	0.01	0.14	0.01	0.15	0.01	0.12	0.01	0.12	0.01
Age group																
18–24	0.22	0.01	0.24	0.01	0.20	0.02	0.26	0.02	0.23	0.02	0.23	0.02	0.22	0.01	0.24	0.01
25–34	0.29	0.01	0.29	0.01	0.33	0.02	0.23	0.02	0.28	0.02	0.26	0.02	0.29	0.01	0.26	0.01
35–44	0.21	0.01	0.24	0.01	0.19	0.02	0.24	0.02	0.22	0.02	0.23	0.02	0.21	0.01	0.24	0.01
45–65	0.29	0.01	0.23	0.01	0.27	0.02	0.28	0.02	0.27	0.02	0.28	0.02	0.28	0.01	0.26	0.01
Caste category																
Scheduled Caste	0.17	0.01	0.18	0.01	0.14	0.02	0.16	0.02	0.12	0.01	0.16	0.01	0.14	0.01	0.17	0.01
Other backward caste	0.5	0.01	0.52	0.01	0.46	0.03	0.44	0.02	0.37	0.02	0.33	0.02	0.43	0.01	0.42	0.01
General	0.23	0.01	0.21	0.01	0.17	0.02	0.17	0.02	0.34	0.02	0.41	0.02	0.28	0.01	0.30	0.01
Brahmin	0.07	0.01	0.06	0.01	0.06	0.01	0.05	0.01	0.02	0.01	0.02	0.00	0.04	0.00	0.04	0.00
Scheduled Tribe	0.03	0.00	0.02	0.00	0.18	0.02	0.17	0.02	0.12	0.01	0.07	0.01	0.09	0.01	0.06	0.00
Other	0.00	0.00	0.00	0.00	0.00	.	0.02	0.01	0.03	0.01	0.02	0.01	0.02	0.00	0.01	0.00
Religion																
Hindu	0.80	0.01	0.84	0.01	0.76	0.02	0.72	0.02	0.88	0.01	0.88	0.01	0.83	0.01	0.84	0.01
Muslim	0.20	0.01	0.16	0.01	0.16	0.02	0.24	0.02	0.09	0.01	0.07	0.01	0.14	0.01	0.13	0.01
Other	0.00	0.00	0.00	0.00	0.08	0.01	0.04	0.01	0.04	0.01	0.05	0.01	0.03	0.00	0.03	0.00
Asset count (mean, out of 5)	2.14	0.04	2.07	0.04	2.50	0.10	2.27	0.08	3.52	0.05	3.69	0.05	2.89	0.03	2.86	0.03
n	1287		1471		362		460		723		750		2372		2681	

*Note*: Observations are adults whose mental health was measured. Weighted proportions and standard errors are shown. For asset count, the mean number of assets in the household (out of 5) is shown. Data are analysed separately by questionnaire to show that random assignment of questionnaires produced statistically similar samples. For the last three ‘Total’ columns, data for all three states are combined, with estimates using pooled weights.

### Response rates and selection into non-response

Table 6 shows marked differences in response rates for the adapted Kessler-6 questionnaire and the adapted SRQ. In each state, the proportion of respondents who answered all mental health questions was statistically significantly lower if assigned the Kessler-6 than if assigned the SRQ. In the pooled sample, the response rate was 82% for the Kessler-6, compared to 94% for the SRQ. In Bihar, the proportion of respondents who responded to all mental health questions was 17 percentage points higher for SRQ than for Kessler-6; in Jharkhand, the difference was 16 percentage points; and in Maharashtra, the difference was 12 percentage points. We note that these disparities are coming mostly from differences in partial response, rather than respondents refusing or being unable to answer all mental health questions.

**Table 6:** Response rates to adapted Kessler-6 and self-reporting questionnaires

Adapted Kessler-6 questionnaire				
State	Answered all questions	Answered some questions	Answered no questions	*n*
Bihar	0.76 [0.72, 0.79]	0.18 [0.15, 0.21]	0.06 [0.05, 0.08]	1676
Jharkhand	0.73 [0.66, 0.79]	0.19 [0.14, 0.25]	0.08 [0.05, 0.14]	466
Maharashtra	0.87 [0.83, 0.90]	0.06 [0.04, 0.10]	0.07 [0.04, 0.10]	820
Total	0.82 [0.79, 0.84]	0.11 [0.10, 0.14]	0.07 [0.05, 0.09]	2964

Adapted self-reporting questionnaire				

State	answered all questions	answered some questions	answered no questions	*n*

Bihar	0.93 [0.91, 0.94]	0.03 [0.02, 0.05]	0.04 [0.03, 0.05]	1619
Jharkhand	0.89 [0.84, 0.93]	0.04 [0.02, 0.07]	0.07 [0.04, 0.11]	500
Maharashtra	0.95 [0.93, 0.97]	0.01 [0.01, 0.03]	0.03 [0.02, 0.05]	784
Total	0.94 [0.93, 0.95]	0.02 [0.02, 0.03]	0.03 [0.03, 0.05]	2903

*Note*: Weighted proportions are shown. 95% confidence intervals are shown in brackets. Sample sizes show the number of respondents considered eligible to respond to a particular questionnaire. A respondent is considered eligible if he/she answered the prior question and was randomly assigned to be asked that questionnaire. For the ‘Total’ rows, data for all three states are combined, with estimates using pooled weights.

Table 7 shows that the Kessler-6 suffers from a greater degree of selective non-response than the SRQ. For the adapted Kessler-6, females, older adults, less educated adults and poorer adults all have statistically significantly higher odds of not responding to the questions. In contrast, none of these characteristics statistically significantly predicts non-response to the SRQ in these models.

**Table 7:** Logit models of selection into non-response to adapted Kessler-6 and self-reporting questionnaires, all states

	Kessler-6	SRQ
Female	1.601^**^	0.826
	(0.279)	(0.207)
Older adult (age 45–65)	1.259	1.029
	(0.246)	(0.277)
Less than secondary education (0–8 years)	2.235^***^	1.079
	(0.332)	(0.253)
Have 2 or fewer assets (of 5)	1.685^**^	1.420
	(0.291)	(0.357)
*N*	2964	2903

*Note*: The table shows odds ratios and standard errors for logit models with only one regressor predicting non-response (complete or partial) to the mental health questions among those who were randomly assigned to answer that set of questions and who had answered the previous section. Standard errors are given in parentheses:

+ *P* < 0.1,

* *P* < 0.05,

** *P* < 0.01,

*** *P* < 0.001.

Data for all three states are combined, and all regressions use pooled weights.

Table 8 presents the marginal effects at the mean of each variable on the probability of non-response (in percentage points) from a logit model that regresses non-response (complete or partial) on the interactions of the four predictors. The conclusion is broadly similar to the one drawn from the associations in Table 7: the Kessler-6 suffers from non-response based on respondent characteristics, but this is far less of a problem for the SRQ.

**Table 8:** Selection into non-response to adapted Kessler-6 and self-reporting questionnaires for all states; marginal effects from logit model interacting all predictors

	Kessler-6	SRQ
Female	0.056*	−0.003
	(0.027)	(0.015)
Older adult (age 45–65)	0.017	0.002
	(0.029)	(0.015)
Less than secondary education (0–8 years)	0.073**	0.001
	(0.024)	(0.013)
Have 2 or fewer assets (of 5)	0.065*	0.023
	(0.025)	(0.014)
*n*	2958	2897

*Note*: The table shows the marginal effects at the mean of each variable on the probability of non-response (in percentage points) from a logit model of non-response (complete or partial) regressed on the interactions of the four predictors, among respondents who were randomly assigned to answer that set of questions and who had answered the previous section. Standard errors are given in parentheses: ^+^*P* < 0.1, *P < 0.05, **P < 0.01, ***P < 0.001. Data for all three states are combined, and the regressions use pooled weights.

### Summary statistics for mental health measurements

**Figure 1:**
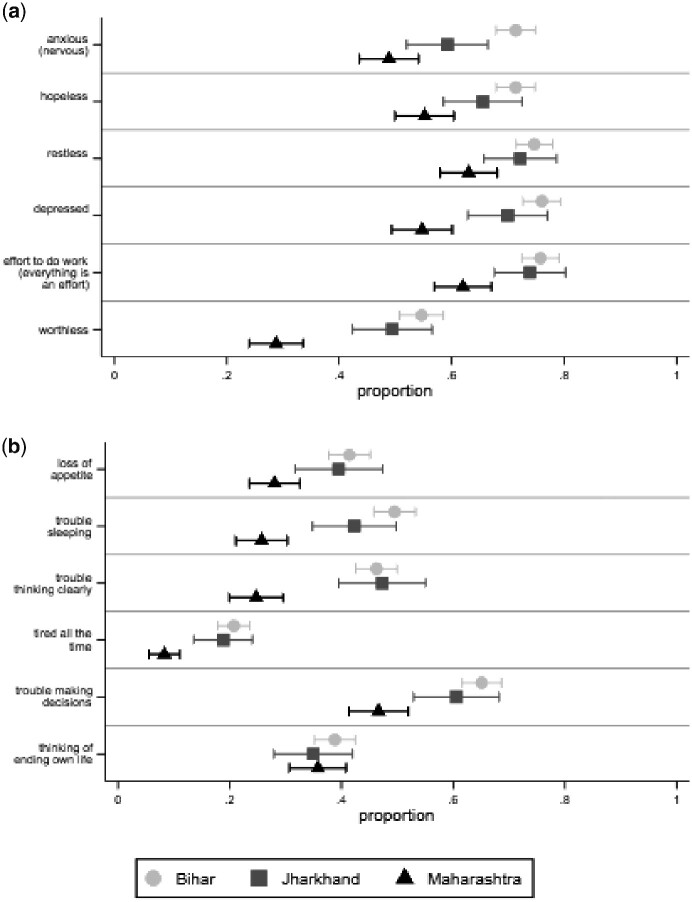
Proportion of respondents who report each symptom in each state. *Note*: The figure shows weighted proportions and 95% CIs for each symptom in each state.

Figure 1 shows the proportion of respondents who report each symptom in each state, with 95% CIs. To compare results from the adapted Kessler-6 questionnaire to results from the adapted SRQ, we collapse the answers to the Kessler-6 questions into a dichotomized variable that takes on ‘1’ if the respondent experienced the symptom ‘sometimes’ or ‘most of the time’ in the 30 days before the survey and ‘0’ if he or she ‘never’ experienced the symptom in the 30 days before the survey. This coding appears to find that respondents to the Kessler-6 have worse average mental health than those who responded to the SRQ. However, levels of mental health cannot be directly compared across the two questionnaires because the coding of responses is not analogous.

One thing that stands out from Figure 1 is that, for almost every symptom, people in Maharashtra report statistically significantly better mental health than people in Jharkhand and Bihar. This makes sense considering the differences across the states in human development.^5^

**Figure 2:**
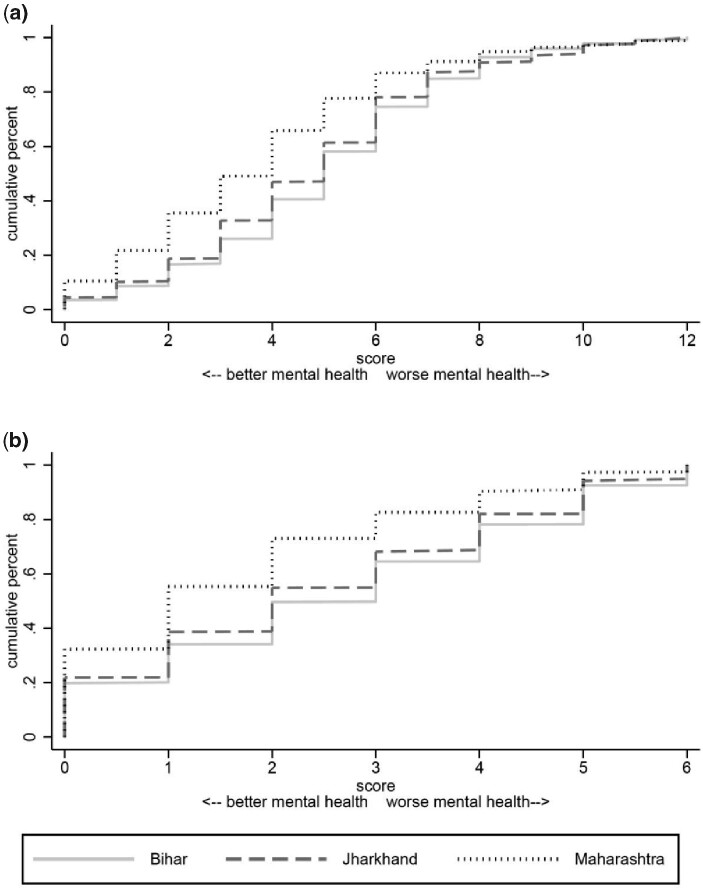
Cumulative distributions of mental health scores, by state. *Note*: A description of how mental health scores are computed is given in the section on ‘Adaptations of Kessler-6 and self-reporting questionnaires’. Response weights are used.

To further investigate differences across states in reported mental health, Figure 2 plots cumulative distribution functions (CDFs) of mental health scores (described above) by state for each questionnaire. The finding that respondents in Maharashtra have better mental health from Figure 1 is also evident in Figure 2. For both the Kessler-6 and the SRQ, the CDF for Maharashtra is always to the left of those for Bihar and Jharkhand. The CDFs for Bihar and Jharkhand are similar for both questionnaires. The Jharkhand CDF stochastically dominates the Bihar CDF for the SRQ, but the CDFs cross for Kessler-6.

### Correlates of poor mental health

**Figure 3:**
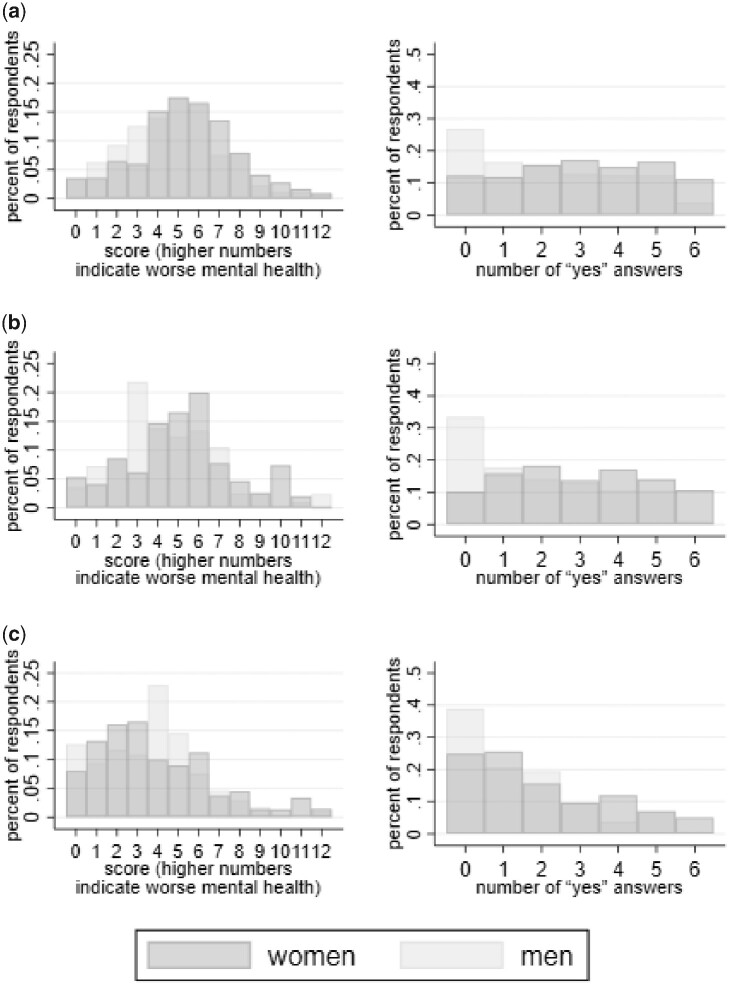
Distributions of mental health scores for adapted Kessler-6 (left column) and self reporting questionnaires (right column), by sex. *Note*: Response weights are used in making the distributions.

We investigate whether the demographic differences in mental health found in the prior literature, described above, are present in the questionnaires we study. Figure 3 shows histograms of mental health score by sex of respondent for each questionnaire in each state. It is visually apparent that in each state, the SRQ classifies women as having worse mental health than men. This is also true of the Kessler-6 questionnaire in Bihar, but differences between men and women are not as visually apparent for the Kessler-6 in Jharkhand and Maharashtra.

Table 9 presents the results of ordered logit regressions of mental health score on demographic characteristics. Coefficients are presented as odds ratios, and standard errors are given in parentheses. Table 9 shows that, with the exception of the Kessler-6 questionnaire in Jharkhand and Maharashtra, being female statistically significantly predicts worse mental health. We note that the magnitude of the coefficient on *female* for Kessler-6 in Jharkhand is similar to the one for Kessler-6 in Bihar, but the sample size is much smaller. Collecting a larger sample of respondents in Jharkhand may have permitted us to identify a statistically significant difference between men’s and women’s mental health using the Kessler-6 questionnaire. Pooled results for both Kessler-6 and SRQ show that being female is a statistically significant predictor of poor mental health.

**Table 9:** Ordered logistic regressions predicting mental health score

	Bihar		Jharkhand		Maharashtra		Total	
	(1)	(2)	(3)	(4)	(5)	(6)	(7)	(8)
	Kessler	SRQ	Kessler	SRQ	Kessler	SRQ	Kessler	SRQ
Female	1.498^*^	1.914^***^	1.499	2.460^***^	1.161	1.733^**^	1.277^+^	1.839^***^
	(0.251)	(0.311)	(0.445)	(0.667)	(0.241)	(0.367)	(0.172)	(0.247)
Age 18–24								
Age 25–34	1.406^+^	0.787	0.958	0.933	1.967^**^	0.977	1.638^**^	0.900
	(0.280)	(0.181)	(0.364)	(0.418)	(0.515)	(0.269)	(0.298)	(0.158)
Age 35–44	1.323	0.996	1.240	1.010	1.585	0.904	1.441	0.953
	(0.270)	(0.223)	(0.480)	(0.442)	(0.537)	(0.253)	(0.328)	(0.170)
Age 45–65	1.685^*^	1.298	0.826	1.156	1.885^*^	0.716	1.655^*^	0.906
	(0.397)	(0.317)	(0.341)	(0.556)	(0.574)	(0.191)	(0.353)	(0.164)
No school								
1–8 years	0.939	1.291	1.164	0.512	1.687	0.853	1.116	0.956
	(0.192)	(0.271)	(0.410)	(0.241)	(0.547)	(0.270)	(0.219)	(0.184)
9–12 years	0.745	0.596^*^	1.184	0.627	1.139	0.691	0.856	0.672^*^
	(0.136)	(0.135)	(0.401)	(0.226)	(0.359)	(0.182)	(0.160)	(0.112)
13+ years	0.513^**^	0.529^*^	1.108	0.617	0.774	0.599^+^	0.622^*^	0.627^*^
	(0.116)	(0.138)	(0.457)	(0.270)	(0.273)	(0.181)	(0.141)	(0.122)
Muslim	1.092	1.289	0.798	0.708	0.817	1.097	1.081	1.245
	(0.303)	(0.276)	(0.563)	(0.273)	(0.283)	(0.307)	(0.247)	(0.203)
F-statistic on caste indicators	6.86	2.03	8.15	8.19	0.97	13.28	3.20	8.18
*P*-value on caste indicators	0.23	0.84	0.09	0.14	0.96	0.02	0.67	0.15
Number of assets (of 5)	0.885^*^	0.824^***^	0.954	0.811^*^	0.837^*^	0.808^**^	0.795^***^	0.764^***^
	(0.0466)	(0.04433)	(0.0774)	(0.0769)	(0.0703)	(0.0648)	(0.0344)	(0.0326)
*n*	1287	1471	362	460	723	750	2372	2681

*Note*: The table shows coefficients as odds ratios from an ordered logistic regression. Standard errors are given in parentheses:

+ *P* < 0.1,

* *P* < 0.05,

** *P* < 0.01,

*** *P* < 0.001.

All regressions use response weights. For models (7) and (8), data for all three states are combined, and regressions use pooled weights.

With the exception of the Kessler-6 in Jharkhand, asset ownership statistically significantly predicts better mental health in all samples, including in the pooled sample. Controlling for assets, people with more schooling typically have lower odds of reporting poor mental health. Across states, the difference between a person with no education and one with 13 or more years of education is more consistently apparent in the SRQ than in the Kessler-6 but is statistically significant for both questionnaires in the pooled sample. Perhaps surprisingly, caste and religion do not predict poor mental health in any of the samples. We discuss these findings below.

## Discussion

This article measures mental health in three states in India using questionnaires adapted for a mobile phone survey. In this section, we reflect on what we learned about measuring population mental health from piloting, adapting and implementing these questionnaires and from analysing results.

Mental health questions were more challenging to ask in the SARI survey than other questions, which measured personal characteristics, social attitudes, and opinions about public policy. Interviewers reported having to spend more effort to avoid hang-ups and other forms of non-response during the mental health questions than on any other question. Mental health questions related to emotions, such as those in the Kessler-6 questionnaire, often required interviewers to give substantial explanations about what the question is asking, which slowed the survey and frustrated respondents.

However, respondents were more forthcoming with answers to questions related to physical symptoms, like those in the adapted SRQ. We hypothesize, but have not tested, that the physical symptoms in the SRQ are more likely to be part of day-to-day conversations than the emotional symptoms in the Kessler-6. In addition, there may be stigma associated with expressing emotional problems. This is consistent with Raguram *et al.*’s (1996) study that finds that patients in Bangalore view reporting depressive symptoms, but not somatic symptoms, as socially disadvantageous because physical symptoms seem similar to illnesses that even people in good mental health could experience. Similarly, Pereira *et al.* (2007) show that women diagnosed with depression expressed their problems primarily through somatic complaints.

Of course, we cannot separate the effect of measuring mental health with questions related to emotion from the fact that the Kessler-6 questionnaire is different in other ways too. It gives three response options, in contrast to the two offered by the SRQ. SARI interviewers reported that three options were difficult for respondents to remember. It is possible more respondents would have answered Kessler-6 questions if they could have responded in a ‘yes’ or ‘no’ format. Doing so, however, would have made this study less comparable with prior studies that use the Kessler-6. Future studies on mental health measurement in LMICs that use mobile phone surveys might usefully test this adaptation of the Kessler-6 questionnaire.

The difficulty that interviewers and respondents had with the Kessler-6 questions is evident in the lower response rates and in the selection into non-response. Respondents’ gender and education play an important role in who completes the Kessler-6 questions. The fact that response rates for Kessler-6 are statistically significantly higher in Maharashtra than in Bihar and Jharkhand is consistent with the fact that respondents in Maharashtra have more education, on average, than respondents in the other states. Considering that women and people with less education are typically vulnerable to worse mental health, it may be advisable to avoid phone survey measurements of population mental health with Kessler-6, or similar multi-response option, emotion-based questionnaires, in LMICs until these populations are found to respond at similar rates to men and more educated people.

The lack of correlation between caste and religion and mental health in the SARI data is perhaps surprising. We do not find that Muslims, who experience social discrimination in India, report worse mental health than Hindus. To our knowledge, there is only one population-level study of mental health among Muslims in India (Gupta and Coffey, 2019), which uses WHO-SAGE data to show that, even accounting for socioeconomic characteristics, Muslims have worse mental health than Hindus. They also find that Scheduled Castes have worse mental health than upper-caste Hindus. The absence of similar associations in SARI may be due to small sample sizes, to selective non-response of Muslim and Scheduled Caste households with the worse mental health, to the modality of the survey (mobile phone survey vs. face-to-face), to the fact that states differed across surveys, or because different questions were asked. The Gupta and Coffey (2019) study analysed two basic questions about whether the respondent had ‘a problem’ with feeling ‘sad, low or depressed’ or ‘worry or anxiety’ in the 30 days before the survey. They report on the severity of these two symptoms among people from different caste and religious groups. The relationship between poor mental health and minority status in India merits further investigation: surveys with larger samples and a wider array of mental health questions are needed to better understand how membership in different caste and religious groups relates to mental health in India.

## Conclusion

In sum, we find that the adapted SRQ, which focuses on physical symptoms instead of emotions, has higher response rates in all three states than the adapted Kessler-6 questionnaire. Both questionnaires classified mental health in Bihar and Jharkhand as markedly worse than in Maharashtra. However, only the SRQ identified women as having worse mental health than men in all three states. The SRQ also more often identified disparities by education.

These findings contribute to health researchers’ efforts to measure levels, trends and disparities in the mental health of populations in two ways. First, they suggest that mobile phone surveys may provide a valuable medium for incorporating mental health measurement into population-level surveys, especially when face-to-face surveys are not possible due to cost or other constraints. Second, they suggest that future research should investigate the merits of asking mental health questions related to physical symptoms rather than emotional ones in similar contexts.

Future research might combine both types of questions into a single questionnaire to see whether the same respondents are classified as having poor mental health by various question types. It is an important and urgent goal to include appropriate mental health questions in nationally representative population health surveys to advocate for better mental health services and track changes in mental health.

## Supplementary Material

czab023_Supp

## Footnotes

1 Although mobile phone interviewers need to be recruited, trained and paid, there are no travel costs, which often constitute a large fraction of the budgets of population-representative surveys in developing countries (Mahfoud *et al.*, 2015).

2 Studies show that results of mental health assessments by phone are highly correlated with in-person assessments (Aneshensel *et al.*, 1982; Fenig *et al.*, 1993; Pinto‐Meza *et al.*, 2005).

3 Three candidate studies for comparison to SARI are the WHO SAGE, the IIPS Youth Study, and the National Mental Health Survey (NMHS) of India. These measure population mental health at the state level, but substantial mismatch in the age and location of the samples make the studies not comparable. The WHO SAGE focuses on older adults, while the IIPS Youth Study interviews respondents 15–29. SARI studies adults 18–65. Although the age range for NMHS is similar, that study focuses on mental disorders rather than mental distress.

4 Caste hierarchies, based on fixed occupational roles with corresponding levels of ritual purity and social ranking (Vaid, 2014), play an important role in Indian society. Untouchability is a severe form of discrimination against Dalits, those of the lowest caste. Traditionally expected to perform tasks considered dirty and impure, they were called ‘untouchable’ as contact with them was considered polluting. Even today, Dalit communities face widespread oppression (Shah *et al.*, 2006).

5 A high fraction of respondents reported that they had thought of ending their life. Although they did not probe, interviewers acknowledged these reports by saying, ‘I am sad to hear you say that. We hope you will begin to feel good again soon’. Since many rural districts lack qualified mental health providers, referrals were not made. We hope to follow up with qualitative work to better understand these responses.
