# Supplementary material for: Measurement of population mental health: evidence from a mobile phone survey in India
Source: Health Policy Plan. 2021 Mar 9;36(5):606–19. doi: 10.1093/heapol/czab023 (PMC8173664; doi:10.1093/heapol/czab023)
Supplement: czab023_Supp [file czab023_supp.zip › Table 1 - SARI sample sizes and response rates, by state.docx]

Table 1. SARI sample sizes and response rates, by state

|  | Sample sizes | | | Response rates |
| --- | --- | --- | --- | --- |
| State | Men | Women | Total | (%) |
| Bihar | 1450 | 1988 | 3438 | 19 |
| Jharkhand | 459 | 550 | 1009 |  |
| Maharashtra | 920 | 746 | 1666 | 25 |
| Total | 2829 | 3284 | 6113 |  |

Note: Survey response rates are calculated as the number of surveys in which a respondent answered at least a third of the questions divided by the number of mobile numbers that were valid (as opposed to nonexistent, switched off, or not available) when they were first called. Response rates for Bihar and Jharkhand cannot be calculated separately because Bihar and Jharkhand mobile numbers are pooled into the same mobile circle by the Telecom Regulatory Authority of India. State of residence is only known for individuals who began the survey, but not for every valid phone number called.
