# Supplementary material for: Measurement of population mental health: evidence from a mobile phone survey in India
Source: Health Policy Plan. 2021 Mar 9;36(5):606–19. doi: 10.1093/heapol/czab023 (PMC8173664; doi:10.1093/heapol/czab023)
Supplement: czab023_Supp [file czab023_supp.zip › Table 2 - Household-level mobile phone ownership, by state.docx]

Table 2. Household-level mobile phone ownership, by state

| State | Urban (%) | Rural (%) | Total (%) |
| --- | --- | --- | --- |
| Bihar | 95 | 89 | 90 |
| Jharkhand | 95 | 80 | 84 |
| Maharashtra | 97 | 86 | 91 |
| Total | 96 | 87 | 90 |

Note: Data source: National Family Health Survey, 2015-16.
