# Supplementary material for: Measurement of population mental health: evidence from a mobile phone survey in India
Source: Health Policy Plan. 2021 Mar 9;36(5):606–19. doi: 10.1093/heapol/czab023 (PMC8173664; doi:10.1093/heapol/czab023)
Supplement: czab023_Supp [file czab023_supp.zip › Table 3 - Characteristics associated with household mobile phone ownership in NFHS-2015, weighted.docx]

Table 3. Characteristics associated with household mobile phone ownership in NFHS-2015, weighted

|  | Bihar | | Jharkhand | | Maharashtra | |
| --- | --- | --- | --- | --- | --- | --- |
|  | does not own phone | owns phone | does not own phone | owns phone | does not own phone | owns phone |
| Caste of household head |  |  |  |  |  |  |
| Proportion Scheduled Caste | 0.299 | 0.197 | 0.169 | 0.137 | 0.213 | 0.174 |
|  | (0.011) | (0.005) | (0.010) | (0.005) | (0.013) | (0.007) |
| Proportion Scheduled Tribe | 0.050 | 0.033 | 0.474 | 0.242 | 0.280 | 0.097 |
|  | (0.012) | (0.002) | (0.016) | (0.009) | (0.022) | (0.005) |
| Proportion OBC | 0.537 | 0.586 | 0.320 | 0.493 | 0.223 | 0.286 |
|  | (0.012) | (0.006) | (0.013) | (0.009) | (0.013) | (0.008) |
| Proportion other | 0.115 | 0.183 | 0.038 | 0.128 | 0.284 | 0.442 |
|  | (0.008) | (0.005) | (0.004) | (0.006) | (0.018) | (0.011) |
| Religion of household head |  |  |  |  |  |  |
| Proportion Hindu | 0.835 | 0.838 | 0.715 | 0.745 | 0.798 | 0.785 |
|  | (0.011) | (0.007) | (0.015) | (0.010) | (0.016) | (0.010) |
| Proportion Muslim | 0.163 | 0.161 | 0.069 | 0.142 | 0.076 | 0.110 |
|  | (0.011) | (0.007) | (0.007) | (0.009) | (0.014) | (0.009) |
| Proportion other | 0.002 | 0.001 | 0.217 | 0.114 | 0.126 | 0.105 |
|  | (0.000) | (0.001) | (0.015) | (0.006) | (0.010) | (0.006) |
| Household electricity |  |  |  |  |  |  |
| Proportion without electricity | 0.686 | 0.385 | 0.454 | 0.150 | 0.276 | 0.056 |
|  | (0.010) | (0.008) | (0.013) | (0.007) | (0.014) | (0.004) |
| Proportion with electricity | 0.314 | 0.615 | 0.546 | 0.850 | 0.724 | 0.944 |
|  | (0.010) | (0.008) | (0.013) | (0.007) | (0.014) | (0.004) |
| Household latrine |  |  |  |  |  |  |
| Proportion that does not use one | 0.895 | 0.648 | 0.922 | 0.660 | 0.640 | 0.261 |
|  | (0.006) | (0.006) | (0.006) | (0.009) | (0.017) | (0.009) |
| Proportion that uses one | 0.105 | 0.352 | 0.077 | 0.340 | 0.360 | 0.739 |
|  | (0.006) | (0.006) | (0.006) | (0.009) | (0.017) | (0.009) |
|  |  |  |  |  |  |  |
| Mean years of household head education | 1.90 | 4.84 | 2.21 | 5.97 | 3.09 | 7.45 |
|  | (0.07) | (0.06) | (0.07) | (0.07) | (0.14) | (0.11) |
|  |  |  |  |  |  |  |

Note: The table shows the distributions of demographic and socioeconomic characteristics among households that do and do no own mobile phones in the NFHS-2015. Survey weights are used to compute the distributions.
