# Supplementary material for: Measurement of population mental health: evidence from a mobile phone survey in India
Source: Health Policy Plan. 2021 Mar 9;36(5):606–19. doi: 10.1093/heapol/czab023 (PMC8173664; doi:10.1093/heapol/czab023)
Supplement: czab023_Supp [file czab023_supp.zip › Table 4 - Original questions asked in Kessler-6 and Self-Reporting Questionnaires.docx]

Table 4. Original questions asked in Kessler-6 and Self-Reporting Questionnaires

| Kessler-6 Questionnaire | Self-Reporting Questionnaire |
| --- | --- |
| 1. About how often during the past 30 days did you feel **nervous** -- would you say all of the time, most of the time, some of the time, a little of the time, or none of the time? 2. About how often during the past 30 days did you feel **hopeless** -- would you say all of the time, most of the time, some of the time, a little of the time, or none of the time? 3. About how often during the past 30 days did you feel **restless or fidgety** -- would you say all of the time, most of the time, some of the time, a little of the time, or none of the time? 4. About how often during the past 30 days did you feel **so depressed that nothing could cheer you up** -- would you say all of the time, most of the time, some of the time, a little of the time, or none of the time? 5. About how often during the past 30 days did you feel **that everything was an effort** -- would you say all of the time, most of the time, some of the time, a little of the time, or none of the time? 6. About how often during the past 30 days did you feel **worthless** -- would you say all of the time, most of the time, some of the time, a little of the time, or none of the time? | 1. Do you often have headaches? 2. Is your appetite poor?* 3. Do you have trouble sleeping?* 4. Are you easily frightened? 5. Do your hands shake? 6. Do you feel nervous, tense, or worried? 7. Is your digestion poor? 8. Do you have trouble thinking clearly?* 9. Do you feel unhappy? 10. Do you cry more than usual? 11. Do you find it difficult to enjoy your daily activities? 12. Do you find it difficult to make decisions?* 13. Is your daily work suffering? 14. Are you unable to play a useful part in life? 15. Have you lost interest in things? 16. Do you feel that you are a worthless person? 17. Has the thought of ending your life been on your mind?* 18. Do you feel tired all the time?* 19. Do you have uncomfortable feelings in your stomach? 20. Are you easily tired? |

Note: Source for Kessler-6 Questionnaire: National Comorbidity Survey: <https://www.hcp.med.harvard.edu/ncs/k6_scales.php>. Source for SRQ: A User’s Guide to the Self-Reporting Questionnaire (Beusenberg and Orley 1994)
