# Supplementary material for: Measurement of population mental health: evidence from a mobile phone survey in India
Source: Health Policy Plan. 2021 Mar 9;36(5):606–19. doi: 10.1093/heapol/czab023 (PMC8173664; doi:10.1093/heapol/czab023)
Supplement: czab023_Supp [file czab023_supp.zip › Table 5 - Summary statistics for predictors of mental health score, by state.docx]

Table 5. Summary statistics for predictors of mental health score, by state

|  | Bihar | | | | Jharkhand | | | | Maharashtra | | | | Total | | | |
| --- | --- | --- | --- | --- | --- | --- | --- | --- | --- | --- | --- | --- | --- | --- | --- | --- |
|  | Kessler | | SRQ | | Kessler | | SRQ | | Kessler | | SRQ | | Kessler | | SRQ | |
|  | prop. | s.e. | prop. | s.e. | prop. | s.e. | prop. | s.e. | prop. | s.e. | prop. | s.e. | prop. | s.e. | prop. | s.e. |
| female | 0.46 | 0.01 | 0.46 | 0.01 | 0.50 | 0.03 | 0.50 | 0.02 | 0.47 | 0.02 | 0.48 | 0.02 | 0.47 | 0.01 | 0.47 | 0.01 |
|  |  |  |  |  |  |  |  |  |  |  |  |  |  |  |  |  |
| **education** |  |  |  |  |  |  |  |  |  |  |  |  |  |  |  |  |
| no schooling | 0.47 | 0.01 | 0.46 | 0.01 | 0.39 | 0.03 | 0.47 | 0.02 | 0.24 | 0.02 | 0.23 | 0.02 | 0.34 | 0.01 | 0.35 | 0.01 |
| 1-8 years schooling | 0.26 | 0.01 | 0.28 | 0.01 | 0.32 | 0.02 | 0.22 | 0.02 | 0.36 | 0.02 | 0.35 | 0.02 | 0.32 | 0.01 | 0.30 | 0.01 |
| 9-12 years schooling | 0.19 | 0.01 | 0.17 | 0.01 | 0.19 | 0.02 | 0.19 | 0.02 | 0.26 | 0.02 | 0.28 | 0.02 | 0.23 | 0.01 | 0.22 | 0.01 |
| 13+ years schooling | 0.08 | 0.01 | 0.09 | 0.01 | 0.10 | 0.02 | 0.12 | 0.01 | 0.14 | 0.01 | 0.15 | 0.01 | 0.12 | 0.01 | 0.12 | 0.01 |
| **age group** |  |  |  |  |  |  |  |  |  |  |  |  |  |  |  |  |
| 18-24 | 0.22 | 0.01 | 0.24 | 0.01 | 0.20 | 0.02 | 0.26 | 0.02 | 0.23 | 0.02 | 0.23 | 0.02 | 0.22 | 0.01 | 0.24 | 0.01 |
| 25-34 | 0.29 | 0.01 | 0.29 | 0.01 | 0.33 | 0.02 | 0.23 | 0.02 | 0.28 | 0.02 | 0.26 | 0.02 | 0.29 | 0.01 | 0.26 | 0.01 |
| 35-44 | 0.21 | 0.01 | 0.24 | 0.01 | 0.19 | 0.02 | 0.24 | 0.02 | 0.22 | 0.02 | 0.23 | 0.02 | 0.21 | 0.01 | 0.24 | 0.01 |
| 45-65 | 0.29 | 0.01 | 0.23 | 0.01 | 0.27 | 0.02 | 0.28 | 0.02 | 0.27 | 0.02 | 0.28 | 0.02 | 0.28 | 0.01 | 0.26 | 0.01 |
| **caste category** |  |  |  |  |  |  |  |  |  |  |  |  |  |  |  |  |
| Scheduled Caste | 0.17 | 0.01 | 0.18 | 0.01 | 0.14 | 0.02 | 0.16 | 0.02 | 0.12 | 0.01 | 0.16 | 0.01 | 0.14 | 0.01 | 0.17 | 0.01 |
| Other Backward Caste | 0.5 | 0.01 | 0.52 | 0.01 | 0.46 | 0.03 | 0.44 | 0.02 | 0.37 | 0.02 | 0.33 | 0.02 | 0.43 | 0.01 | 0.42 | 0.01 |
| General | 0.23 | 0.01 | 0.21 | 0.01 | 0.17 | 0.02 | 0.17 | 0.02 | 0.34 | 0.02 | 0.41 | 0.02 | 0.28 | 0.01 | 0.30 | 0.01 |
| Brahmin | 0.07 | 0.01 | 0.06 | 0.01 | 0.06 | 0.01 | 0.05 | 0.01 | 0.02 | 0.01 | 0.02 | 0.00 | 0.04 | 0.00 | 0.04 | 0.00 |
| Scheduled Tribe | 0.03 | 0.00 | 0.02 | 0.00 | 0.18 | 0.02 | 0.17 | 0.02 | 0.12 | 0.01 | 0.07 | 0.01 | 0.09 | 0.01 | 0.06 | 0.00 |
| Other | 0.00 | 0.00 | 0.00 | 0.00 | 0.00 | . | 0.02 | 0.01 | 0.03 | 0.01 | 0.02 | 0.01 | 0.02 | 0.00 | 0.01 | 0.00 |
| **religion** |  |  |  |  |  |  |  |  |  |  |  |  |  |  |  |  |
| Hindu | 0.80 | 0.01 | 0.84 | 0.01 | 0.76 | 0.02 | 0.72 | 0.02 | 0.88 | 0.01 | 0.88 | 0.01 | 0.83 | 0.01 | 0.84 | 0.01 |
| Muslim | 0.20 | 0.01 | 0.16 | 0.01 | 0.16 | 0.02 | 0.24 | 0.02 | 0.09 | 0.01 | 0.07 | 0.01 | 0.14 | 0.01 | 0.13 | 0.01 |
| Other | 0.00 | 0.00 | 0.00 | 0.00 | 0.08 | 0.01 | 0.04 | 0.01 | 0.04 | 0.01 | 0.05 | 0.01 | 0.03 | 0.00 | 0.03 | 0.00 |
|  |  |  |  |  |  |  |  |  |  |  |  |  |  |  |  |  |
| asset count (mean, out of 5) | 2.14 | 0.04 | 2.07 | 0.04 | 2.50 | 0.10 | 2.27 | 0.08 | 3.52 | 0.05 | 3.69 | 0.05 | 2.89 | 0.03 | 2.86 | 0.03 |
| n | 1287 |  | 1471 |  | 362 |  | 460 |  | 723 |  | 750 |  | 2372 |  | 2681 |  |

Note: Observations are adults whose mental health was measured. Weighted proportions and standard errors are shown. For asset count, the mean number of assets in the household (out of 5) is shown. Data are analyzed separately by questionnaire to show that random assignment of questionnaires produced statistically similar samples. For the last three ‘Total’ columns, data for all three states are combined, with estimates using pooled weights.
