# Supplementary material for: Measurement of population mental health: evidence from a mobile phone survey in India
Source: Health Policy Plan. 2021 Mar 9;36(5):606–19. doi: 10.1093/heapol/czab023 (PMC8173664; doi:10.1093/heapol/czab023)
Supplement: czab023_Supp [file czab023_supp.zip › Table 6 - Response rates to adapted Kessler-6 and Self-Reporting Questionnaires.docx]

Table 6. Response rates to adapted Kessler-6 and Self-Reporting Questionnaires

|  | **adapted Kessler-6 questionnaire** | | | |
| --- | --- | --- | --- | --- |
| **state** | answered all questions | answered some questions | answered no questions | *n* |
| Bihar | 0.76 [0.72, 0.79] | 0.18 [0.15, 0.21] | 0.06 [0.05, 0.08] | 1676 |
| Jharkhand | 0.73 [ 0.66, 0.79] | 0.19 [0.14, 0.25] | 0.08 [0.05, 0.14] | 466 |
| Maharashtra | 0.87 [0.83, 0.90] | 0.06 [0.04, 0.10] | 0.07 [0.04, 0.10] | 820 |
| Total | 0.82 [0.79, 0.84] | 0.11 [0.10, 0.14] | 0.07 [0.05, 0.09] | 2964 |
|  |  |  |  |  |
|  | **adapted Self-Reporting Questionnaire** | | | |
| **state** | answered all questions | answered some questions | answered no questions | *n* |
| Bihar | 0.93 [0.91, 0.94] | 0.03 [0.02, 0.05] | 0.04 [0.03, 0.05] | 1619 |
| Jharkhand | 0.89 [0.84, 0.93] | 0.04 [0.02, 0.07] | 0.07 [0.04, 0.11] | 500 |
| Maharashtra | 0.95 [0.93, 0.97] | 0.01 [0.01, 0.03] | 0.03 [0.02, 0.05] | 784 |
| Total | 0.94 [0.93, 0.95] | 0.02 [0.02, 0.03] | 0.03 [0.03, 0.05] | 2903 |

Note: Weighted proportions are shown. 95% confidence intervals are shown in brackets. Sample sizes show the number of respondents considered eligible to respond to a particular questionnaire. A respondent is considered eligible if he/she answered the prior question and was randomly assigned to be asked that questionnaire. For the ‘Total’ rows, data for all three states are combined, with estimates using pooled weights.
