# Supplementary material for: Measurement of population mental health: evidence from a mobile phone survey in India
Source: Health Policy Plan. 2021 Mar 9;36(5):606–19. doi: 10.1093/heapol/czab023 (PMC8173664; doi:10.1093/heapol/czab023)
Supplement: czab023_Supp [file czab023_supp.zip › Table 9 - Ordered logistic regressions predicting mental health score.docx]

Table 9. Ordered logistic regressions predicting mental health score

|  | Bihar | | Jharkhand | | Maharashtra | | Total | |
| --- | --- | --- | --- | --- | --- | --- | --- | --- |
|  | (1) | (2) | (3) | (4) | (5) | (6) | (7) | (8) |
|  | Kessler | SRQ | Kessler | SRQ | Kessler | SRQ | Kessler | SRQ |
| female | 1.498* | 1.914*** | 1.499 | 2.460*** | 1.161 | 1.733** | 1.277+ | 1.839*** |
|  | (0.251) | (0.311) | (0.445) | (0.667) | (0.241) | (0.367) | (0.172) | (0.247) |
| age 18-24 |  |  |  |  |  |  |  |  |
| age 25-34 | 1.406+ | 0.787 | 0.958 | 0.933 | 1.967** | 0.977 | 1.638** | 0.900 |
|  | (0.280) | (0.181) | (0.364) | (0.418) | (0.515) | (0.269) | (0.298) | (0.158) |
| age 35-44 | 1.323 | 0.996 | 1.240 | 1.010 | 1.585 | 0.904 | 1.441 | 0.953 |
|  | (0.270) | (0.223) | (0.480) | (0.442) | (0.537) | (0.253) | (0.328) | (0.170) |
| age 45-65 | 1.685* | 1.298 | 0.826 | 1.156 | 1.885* | 0.716 | 1.655* | 0.906 |
|  | (0.397) | (0.317) | (0.341) | (0.556) | (0.574) | (0.191) | (0.353) | (0.164) |
| no school |  |  |  |  |  |  |  |  |
| 1-8 years | 0.939 | 1.291 | 1.164 | 0.512 | 1.687 | 0.853 | 1.116 | 0.956 |
|  | (0.192) | (0.271) | (0.410) | (0.241) | (0.547) | (0.270) | (0.219) | (0.184) |
| 9-12 years | 0.745 | 0.596* | 1.184 | 0.627 | 1.139 | 0.691 | 0.856 | 0.672* |
|  | (0.136) | (0.135) | (0.401) | (0.226) | (0.359) | (0.182) | (0.160) | (0.112) |
| 13+ years | 0.513** | 0.529* | 1.108 | 0.617 | 0.774 | 0.599+ | 0.622* | 0.627* |
|  | (0.116) | (0.138) | (0.457) | (0.270) | (0.273) | (0.181) | (0.141) | (0.122) |
| Muslim | 1.092 | 1.289 | 0.798 | 0.708 | 0.817 | 1.097 | 1.081 | 1.245 |
|  | (0.303) | (0.276) | (0.563) | (0.273) | (0.283) | (0.307) | (0.247) | (0.203) |
| F-statistic on caste indicators | 6.86 | 2.03 | 8.15 | 8.19 | 0.97 | 13.28 | 3.20 | 8.18 |
| p-value on caste indicators | 0.23 | 0.84 | 0.09 | 0.14 | 0.96 | 0.02 | 0.67 | 0.15 |
| number of assets (of 5) | 0.885* | 0.824*** | 0.954 | 0.811* | 0.837* | 0.808** | 0.795*** | 0.764*** |
|  | (0.0466) | (0.04433) | (0.0774) | (0.0769) | (0.0703) | (0.0648) | (0.0344) | (0.0326) |
| n | 1287 | 1471 | 362 | 460 | 723 | 750 | 2372 | 2681 |

Note: Table shows coefficients as odds ratios from an ordered logistic regression. Standard errors are given in parentheses: + p<0.1 * p<0.05 ** p<0.01 *** p<0.001. All regressions use response weights. For models (7) and (8), data for all three states are combined, and regressions use pooled weights.
