# Supplementary material for: Measurement of population mental health: evidence from a mobile phone survey in India
Source: Health Policy Plan. 2021 Mar 9;36(5):606–19. doi: 10.1093/heapol/czab023 (PMC8173664; doi:10.1093/heapol/czab023)
Supplement: czab023_Supp [file czab023_supp.zip › response to reviewers.docx]

**Response to reviewers for “Measurement of population mental health: Evidence from a mobile phone survey in India" (HEAPOL-2020-Aug-0653)**

We would like to thank the reviewers for these helpful comments, and the editor for the opportunity to revise the paper. Below, we describe how we have responded to each comment. We believe that, thanks to the thoughtful comments, the paper is now substantially improved. The comments are reproduced below (and indented) for ease of re-review.

Reviewer: 1

The study is well written and focuses on an important research topic: understanding and measuring common mental health disorders/problems in LMICs. The research is thus topical and has potential to contribute to the field. However, I see several limitations in the study design and in the conducted analysis which need to be addressed.

(1) The main aim of the analysis is unclear to me. Is it to test whether mental health measured via mobile phone interviews differs to in-person interviews? If so, I would have expected this to be reflected in the design of the study, e.g. by randomising phone interviews and in-person interviews. Alternatively, in the analysis by at least comparing the findings from the mobile phone interviews to existing mental health measures of similar populations in the same context. Neither was done.

(2) Is it about understanding if the modified Kessler-Scale (K6) and Self-Reporting Questionnaire (SRQ) differ from existing mental health measures? If so, I would have expected a comparison of an unmodified and modified version of either. In contrast, the authors designed modified K6 scales and SRQ and compare their performance with each other. It would be advisable to identify existing data on mental health from India or other LMIC context and to compare the modified to the respective unmodified versions. In short, the study design and analysis missed to identify the all-important comparator to make a real assessment and thus to fill an important research gap in mental health research.

Reviewer 1 makes the important point that the set-up of the comparisons made in the paper is not typical: our paper does not compare measurements of mental health from phone interviews to in-person interviews, nor does it compare modified and unmodified version of the K6 and SRQ questionnaires. Instead, it describes the experience of modifying the K6 and SRQ for use in population-representative phone surveys in India; shows that even simplified versions of the K6 has selective non-response in these populations; and concludes that a carefully selected set of questions, such as the modified SRQ, can nevertheless be usefully employed to measure population mental health in a phone survey in a poor country population with low average education.

The reviewer’s comment suggests that we could have been clearer about what the paper is and is not able to accomplish, and why what it does accomplish is useful. We have substantially revised the introduction, evidence from prior studies, and conclusion sections to add these clarifications. In particular, we now discuss how there are only three studies -- the WHO SAGE, the IIPS Youth Study, and the National Mental Health Survey of India -- that measure population mental health at the state level. Unfortunately, it does not make sense to compare our results to the results of these face-to-face surveys for reasons which we now describe in the manuscript.

To summarize the reasons: In the case of the IIPS Youth Survey, respondents were between the ages of 15 and 29; respondents to the SARI survey were between the ages of 18 and 65. In the case of the WHO SAGE, which surveyed six states, the age ranges match, but the only overlapping state in the two studies is Maharashtra. We do not compare the results for Maharashtra because the same questions were not asked and because WHO SAGE and SARI were done 10 years apart. (When piloting the mental health questions, we considered a design in which we asked the same questions as the WHO SAGE over the phone. Yet we found that the questions from that face-to-face survey were not well-suited to phone interviews. Most of the mental health questions in WHO SAGE had five response options, which phone survey respondents could not keep track of. We decided to focus our efforts on creating and testing useful phone survey questions, rather than testing questions used in prior face to face surveys more extensively in the phone survey context. Finally, we do not make comparisons to the 2015-16 National Mental Health Survey of India because the focus of the NMHS was on measuring more severe mental disorders using the Mini International Neuro-Psychiatric Inventory as well as screening for epilepsy, intellectual disability, and autism spectrum disorders. The focus of the SARI survey was on measuring mental distress at the population level. Further, the only overlapping state is Jharkhand.

Despite the fact that we are not able to make meaningful quantitative comparisons of the SARI phone survey data to other data sources, we believe that the paper nevertheless presents useful results for other researchers to build upon. For example, it serves as a proof-of-concept that mental health questions can be incorporated into phone surveys if the questions and response options are simple enough. This is especially important as the COVID-19 pandemic has led researchers to seek alternatives to in-person surveys. This study also provides useful insights for choosing mental health questions in poor country populations with low average education. Among them are that piloting is needed to ensure that attempts to measure mental health do not lead to non-response and that questions related to physical symptoms rather than emotions are easier to answer. We also hope that these data will serve as a baseline for future research tracking population mental health in the states of Bihar, Jharkhand, and Maharashtra. In particular, by showing that mental health screening questionnaires can work in relatively inexpensive phone surveys, we hope that governments will find it more feasible to track mental health on a regular basis.

(2) A few analytical approaches would need some tweaking. Firstly, I would suggest using a logit/probit model in the estimation of response-selection instead of a linear probability model. Accordingly, reporting marginal effects which facilitate the interpretation if the coefficients. I would suggest to the authors to estimate the model including all covariates jointly, not as separate singular estimator equations. Secondly, a helpful approach to understand associations of K6 with SRQ would be to understand correlations at different levels of the measures. Using the ordered logit model, the authors would use only the pooled model with for example the K6 as outcome and regress the K6 on control variables and on all levels of the SRQ. Then, the authors could show predicted marginal effects of each level (i.e. value) of SRQ at different K6 level (i.e. value). This approach would then help to understand if lower levels of SRQ predict lower levels of K6 etc.

Thank you for this suggestion. We updated the table (now Table 7) to use logit models instead of the OLS model. We would like to include the results of regressions with single variables in order that policymakers who might be reading the article would be able to understand the simple associations between each of the variables and non-response. We think it is the most accessible way to present the idea that less advantaged people are under-represented in responding to the K6. That said, we agree with the reviewer that the marginal effects estimates from an interacted model would enhance our results by showing the joint associations of these variables with non-response. Therefore, we added a new Table 8 presenting marginal effects from a fully interacted model.

Unfortunately, we will not be able to do the second analysis that the reviewer recommend. That is because each respondent only answered one set of mental health questions. We are able to make comparisons across the SRQ and K6 because the questionnaire was randomly assigned. But we would not be able to do the analysis that was recommended because they are not measured for the same person. We’ve clarified this in the text, and mention it as a useful analysis of future datasets: “Future research might combine both types of questions into a single questionnaire to see whether the same respondents are classified as having poor mental health by various question types.”

(3) The findings of better mental health outcomes in Maharashtra and worse mental health outcomes in Bihar and Jharkhand contrast findings from the latest burden of mental health study in India by the WHO (Lancet, 2020), where common mental health disorders such as depression and anxiety disorders were found to be more prevalent in Maharashtra and less prevalent in Bihar and Jharkhand. As such findings from the presented analysis are not consistent with the existing literature (authors note their findings are consistent with the literature p.24, 24ff). I would suggest to the authors to elaborate in the discussion on why they have found different findings for population mental health and use reference. Are the authors confident in their findings? A hypothesis could be that the low response rate lead to an overrepresentation of individuals with good mental health in Maharashtra casting then doubt on the study finding’s internal validity.

Thank you for calling our attention to this study. We agree that we should have cited it and discussed it in our paper. We think that one reason for the difference between this study’s finding of better mental health in Maharashtra as compared to Bihar and Jharkhand could be because the Lancet study focused on mental disorders (depressive disorders, anxiety disorders, schizophrenia, bipolar disorder, idiopathic developmental intellectual disability (IDID), conduct disorder, autism spectrum disorders, eating disorders, attention-deficit hyperactivity disorder) whereas our study measured mental distress using screening tools. Although we do not know for sure, we suspect that the fact that states with higher development had a higher prevalence of mental disorders in the Lancet study could be because capacity for diagnosis of mental disorders is higher in these states. That is also consistent with the finding that states with greater increases in development over the period studied by the Lancet paper had greater increases in mental disorders. We now discuss this paper, as well as the difference between studying mental disorder and mental distress, in the text. (We do not think that non-response could drive our results: response rates for SRQ are not statistically significantly different across states, but the SRQ shows less mental distress in Maharashtra than Bihar and Jharkhand.)

(4) It is not clear to me if the survey weights are used in all estimations and in the descriptive statistics.

Yes, survey weights are used in all computations. We have clarified this in the text.

(5) X-axis titles of figure 2 incorrect, should be “ Better mental health; Worse mental health”

Thank you for catching this! We fixed it.

(6) The authors note on page 8 “The SARI builds a representative sample…”. A representative sample of what? Looking at the age-group variables I wonder whether the SARI-study is limited to the working age-population as age-groups range from 18-24;..;60-65? What about the older population? More information about the representativeness, about where in the three states the SARI was conducted (rural/urban), when the surveys where conducted and whether the SARI is a one-off cross-sectional or repeated would need to be added. Was the study designed using clustering? This adds to another question, what can the authors say about the external validity of their study findings? Do findings apply to the population living in the three states, in other Indian states, or outside of India? The latter points need to be addressed in the discussion alongside the low response rates.

Thank you for pointing out this oversight. We have added information on the SARI sample to this part of the paper. For each of the three states, our findings are representative of adults 18 – 65 (both urban and rural) living in households with a mobile phone. The SARI survey was previously conducted in other states and cities of India, including Uttar Pradesh, Mumbai, Delhi, and Rajasthan, but those surveys did not include mental health questions. To date, the SARI surveys have been done only once in each place. In the future, the survey may be repeated in the same place, but these would be repeated cross sections rather than panel surveys. The design is not clustered because we were calling randomly generated phone numbers.

(7) The statement on page 2 “measurement of mental health in population health surveys is uncommon outside of high-income countries” is exaggerated. I fully agree with the notation that mental health requires more attention, especially in the context of LMICS. However, I am myself aware of several surveys on populations from LMICs that have included measures of mental health, such as the NIDS in South Africa, the MLFHS in Malawi, the MXFLS in Mexico, the IFLS in Indonesia and numerous sister studies of the ELSA in China, Indonesia, Brazil, India, Mexico, Thailand etc.

This is a good point. The sentence now reads “less common.”

(8) Do the authors have information about those individuals that did not respond the questionnaire at all, i.e. cast, income-levels etc? Would be good to present. Further, do the authors have information on who the 10% among households without telephone access are (table 2)? This is quite important for marginalised populations, i.e. poorer, lower casts, possibly mentally ill, as those individuals will be likely excluded from phone surveys and their mental health not considered.

Unfortunately, we were not able to collect information on demographic characteristics from those who did not respond to the questionnaire at all. As part of the quality checks on the data and as part of constructing the weights, we compared the population of respondents to the state populations from Census 2011. The revised version of the paper cites a report about the SARI methodology that elaborates on these comparisons. In addition, we have included a new Table 3 that compares households that own a mobile phone in NFHS-2015 with those that do not in each state, for the variables that the reviewer recommends. More marginalized households are less likely to own phones, which may lead us to underestimate mental distress. We note this in the text. We also note that mobile phone ownership has been growing quickly in India, so that the extent of undercoverage of marginalized households is less than it was when NFHS-2015 was collected.

(9) The authors use a sub-set of mental health determinants. Does the SARI provide any further information about for example alcohol or substance (ab)use, physical health, household characteristics (marriage status, household size, number of dependent children), maternal/paternal mental health history, access to social security programmes, access to health care, household income, employment status, neighbourhood characteristics, or housing quality? If so, it would be helpful to include any available determinants as additional covariates. This may also help explaining the findings from cast and religion with mental health (i.e. omitted variable bias).

Unfortunately, SARI only collected a limited number of covariates, so we are not able to add the analyses the reviewer recommends. However, we have added a more detailed discussion of the caste and religion findings to the end of the paper. Reviewer 2 also commented that it would be useful to know more about these findings.

Reviewer: 2

Comments to the Author

This paper addresses an important topic in Global Mental Health, and is particularly pertinent to countries like India where the need to measure mental health at the population through the use of mobile phones which otherwise is very resource intensive.

The research addresses important questions in the field and is methodologically rigorous. This would make manuscript once the following queries are addressed.

1. The first and the most important thing I noticed is lacking in the last few sentences in the introduction section and overall in the discussion is that the authors claim that how the discussion justifies the hypothesis that adapted SRQ, can be usefully employed to measure population mental health using phone surveys. I would suggest to prune down this sentence and discussion overall to incorporate understanding of contextual factors which may affect the actual implementation of using adapted SRQs in real practice.

Thank you for this suggestion and for your close attention to our conclusions about using the adapted SRQ in practice. We have replaced this text with your suggestion in mind. The new text is streamlined while also emphasizing the important practical issues of implementing the adapted SRQ in this context.

2. Overall some of the sentences were also sometimes a little imprecise, and in some cases the meaning isn't very clear. The manuscript needs to be carefully proof read through by one of the authors to correct these errors.

Thank you for this comment. We have proofread carefully, caught several errors, and note changes in track changes.

3. The broad messages in the paper and the methodology used, especially to calculate response rate and non- response rate are sensible and applaudable.

Thank you!

4. This is one of the few studies which attempts to find out the association between poor mental health and minority status in India, and is therefore crucial to move the mental health rhetoric from symptom management to broader social issues.

Authors found that there is lack of association between caste, religion and mental health and then justify that this may be due to smaller sample sizes. Different question type may have affected this analysis is also stated, I wonder if the authors could expand on this in the discussion section.

The revised version of the manuscript expands on the discussion of disparities in mental health between general caste Hindu and socially marginalized populations. It now reads:

The lack of correlation between caste and religion and mental health in the SARI data is perhaps surprising. We do not find that Muslims, who experience social discrimination in India, report worse mental health than Hindus. To our knowledge, there is only one population-level study of mental health among Muslims in India (Gupta and Coffey 2019), which uses WHO-SAGE data to show that, even accounting for socioeconomic characteristics, Muslims have worse mental health than Hindus. They also find that Scheduled Castes have worse mental health than upper-caste Hindus. The absence of similar associations in SARI may be due to small sample sizes, to selective non-response of Muslim and SC households with the worse mental health, to the modality of the survey (mobile phone survey vs. face-to-face), to the fact that states differed across surveys, or because different questions were asked. The Gupta and Coffey (2019) study analyzed two basic questions about whether the respondent had “a problem” with feeling “sad, low, or depressed” or “worry or anxiety” in the 30 days before the survey. They report on the severity of these two symptoms among people from different caste and religious groups. The relationship between poor mental health and minority status in India merits further investigation: surveys with larger samples and a wider array of mental health questions are needed to better understand how membership in different caste and religious groups relates to mental health in India.
